# Does mandibular advancement with clear aligners have the same skeletal and dentoalveolar effects as traditional functional appliances?

**DOI:** 10.1186/s12903-023-02709-5

**Published:** 2023-02-02

**Authors:** Yanqi Wu, Qian Yu, Yunhui Xia, Bo Wang, Siyue Chen, Kaijun Gu, Bojun Zhang, Min Zhu

**Affiliations:** 1 Department of Oral and Cranio-Maxillofacial Surgery, Shanghai Ninth People’s Hospital, Shanghai Jiao Tong University School of Medicine, 639 Zhizaoju Road, Shanghai, 200011 China; 2 College of Stomatology, Shanghai Jiao Tong University, Shanghai, 200011 China; 3 National Center for Stomatology, National Clinical Research Center for Oral Diseases, Shanghai, 200011 China; 4 Shanghai Key Laboratory of Stomatology, Shanghai Research Institute of Stomatology, Shanghai, 200011 China; 5 Department of Pediatric Dentistry, Shanghai Xuhui District Dental Center, Shanghai, 200032 China

**Keywords:** Class II malocclusion, Clear aligners, Functional appliances, Cephalometric, Johnston’s Pitchfork Analysis

## Abstract

**Background:**

The study aimed to compare the dentoskeletal effects of Vanbeek Activator, Herbst, Twin-Block and Mandibular Advancement with clear aligners in children with skeletal Class II malocclusions.

**Methods:**

A sample with sixty-three patients (37 males, 26 females) was included and divided into untreated control group (C, n = 12), Vanbeek Activator group (V, n = 14), Herbst group (H, n = 11), Twin-Block group (TB, n = 12) and MA group (MA, n = 14). Cephalometric analysis and Johnston Pitchfork analysis were performed to quantify the skeletal and dentoalveolar components in molar relationship and overjet correction. Compare the differences of cephalometric data and Johnston-analysis data.

**Results:**

The treatment changes showed significant differences in SNB, FH-NP, NA-PA, Co-Go, Co-Pog, ANB, lower facial height ratio, U1-PP, U6-PP, L1-MP and U1-L1. All the appliances improved overjet relationships significantly (Vanbeek, Herbst, Twin-Block and MA were 2.77 mm, 5.53 mm, 4.73 mm and 3.66 mm respectively) with significant retraction of maxillary incisors. The lower incisor displacement of group V and MA was negative, while that of group H and TB was positive and there were significant differences. Molar relationships were also improved by 3.45 mm, 6.85 mm, 3.48 mm and 0.92 mm for Vanbeek, Herbst, Twin-Block and MA. Mandible displacement showed a trend of group H > TB > V > MA. The displacement of maxillary molars in group H was greater than that in group C, TB and MA, and that of mandibular ones was greater than that in group C, V and MA, significantly. Herbst, Twin-Block and MA have more significant dentoalveolar effect than Vanbeek, while Vanbeek has more skeletal effect than the others especially in restraining maxillary growth.

**Conclusions:**

Four appliances are all effective in mandibular advancement, modification of class II molar relationship and deep overjet, with unavoidable increase in lower facial ratio. Vanbeek Activator has the most skeletal effects. Vanbeek and MA have a good control of mandibular incisors while more compensatory lower incisors proclination in Herbst and Twin-Block. Herbst has greater maxillary molar distalization. MA allows aligning and leveling meanwhile leading the mandible forward.

## Introduction

Skeletal Class II malocclusion is a common orthodontic problem, mostly the mechanism of which is hypoplasia or retraction of the mandible. Differing from the epiphyseal plates of long bones, the condylar cartilage responds positively to mechanical stimulation [1]. Therefore, for such adolescent patients, the ideal treatment method is enhancing the growth and development potential of the condyle to correct the sagittal dysregulation of both jaws and reduce the possibility of orthognathic surgery in adulthood. Functional appliance has been used to correct skeletal class II malocclusion with a history over 100 years, since Robin and Andresen found it effective in stimulating mandibular growth [2].

Various appliances, such as Activator, Herbst and Twin-Block, would produce a combined effect of skeletal and dentoalveolar changes, because they are supported directly by teeth instead of bone. Dentoalveolar effects are ascribed to retrusion of upper anterior incisors and protrusion of lower incisors [3–5], while skeletal effects are well known as the expected correction, such as mandibular advancement and elongation. Mandible clockwise rotation and increase of lower facial height often occurred meanwhile, which is detrimental to long-face patients. Headgear is always believed as one of the most effective methods for maxilla growth inhibition and vertical control, Vanbeek Activator, as a modification of Headgear-Activator, placing the extraoral bow directly into the plastic base and moving it forward to canine and first premolar region from molar region grew out of that. Vanbeek Activator combined with high-pull headgear could actualize vertical dimension and occlusal plane control [6].

In recent years an invisible appliance called Invisalign Mandibular Advancement (MA) implemented by Align Technology™ has been gradually put into clinical use, which could align teeth while repositioning the lower jaw to the forward extension with a “precision wing brace”. Compared with traditional appliances, MA has good aesthetics, high wearing comfort, higher accuracy and can complete orthopedic and orthodontic treatment at the same time. MA appears to be effective in the treatment of class II malocclusions with mandibular retrusion [7–9]. Like functional appliances, the correction of occlusion relationships is combined skeletal and dental effects, and Sabouni et.al found that the skeletal changes may be minor [10].

The actual effect of functional appliances has always been a controversial topic. Some previous studies focused on common traditional functional appliances, but limited studies were based on MA. Therefore, this retrospective study aimed to deeply compare the skeletal and dentoalveolar effects of MA, Vanbeek Activator, Herbst and Twin-Block in children with Skeletal Class II Malocclusion.

## Materials and methods

### Subjects

In this retrospective study, a sample of 63 patients (Table 1) was collected from the Class II patients who were treated by Dr. Min Zhu. The inclusion criteria included skeletal class II malocclusion with ANB greater than 4°; increased overjet greater than 5 mm; Angle class II molar and canine relationship; cervical vertebral maturation (CVM) CVM2 and no history of orthodontic treatment before. All the cases were divided into Vanbeek Activator group (V, n = 14), Herbst group (H, n = 11), Twin-Block group (TB, n = 12) and MA group (MA, n = 14) according to the therapeutic options. And 12 untreated subjects were selected as control group to assess the effect of growth.

**Table 1:** Sample characteristics

Group	Boys(n)	Girls(n)	Age at T1 (y)	Interval (T2 − T1) (m)
Vanbeek	7	7	10.71 ± 1.44	7.28 ± 2.30
Herbst	4	7	11.55 ± 0.69	10.18 ± 3.06
Twin-Block	7	5	11.00 ± 1.04	10.16 ± 5.46
MA	12	2	12.11 ± 1.16	22.84 ± 8.98
Control	7	5	10.41 ± 0.90	10.25 ± 3.74

### Cephalometric method

**Fig. 1:**
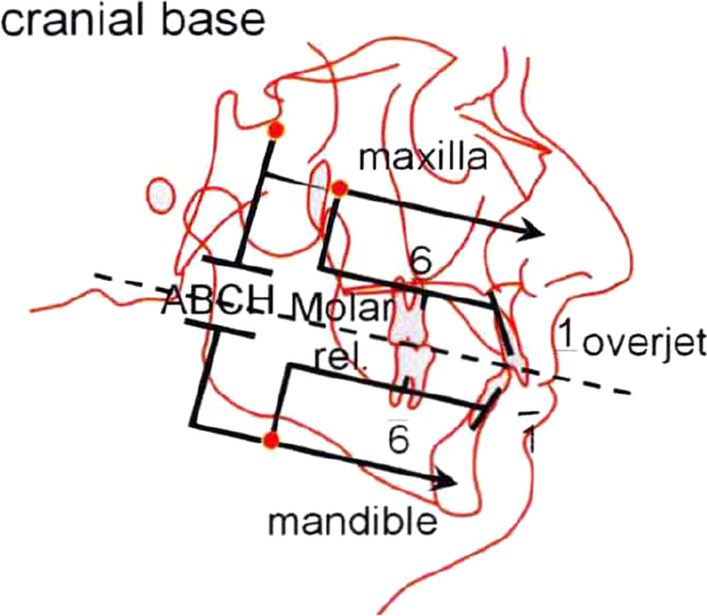
Johnston’s Pitchfork Analysis. Max: maxilla; ABCH: relative AP movement between mandible and maxilla; Mand: mandible; U1: upper incisors; L1: lower incisors; U6: upper molars; L6: lower molars. Max described the AP movement of cranial base landmark, which represented the change of maxilla. Apical base change (ABCH) described the relative AP movement between mandible and maxilla, and Mand described the AP movement of mandible which was the algebraic sum of Max and ABCH. Similarly, U1, L1, U6 and L6 described the AP movements of incisors and molars. All the changes in position benefit to correct class II malocclusion were counted as plus signs while those worsen the malocclusion were counted as minus signs(11)

All the included subjects have taken lateral cephalograms before (T1) and immediately after treatment (T2). Every radiograph was traced 3 times on different times by the same examiner using Dolphin Imaging software. 20 dentoalveolar measurements were calculated, including SNA, SNB, ANB, mandibular plane angle (MP-FH), facial angle (FH-NP), angle of convexity (NA-PA), Co-Go, Go-Pog, Y-Axis angle, lower facial height ratio, vertical ratio (ALFH/PLFH), P-A face height (S-Go/N-Me), maxillary incisor angle (U1-SN), mandibular incisor angle (L1-MP), occlusal plane angle (OP-FH), inter incisal angle (U1-L1), maxillary incisor to palatal plane (U1-PP), maxillary molar to palatal plane (U6-PP). A simplified Johnston’s Pitchfork diagram was applied to analyze dental and skeletal movements (Fig. 1).

### Statistical analysis

All the measurements were transferred to SPSS Statistics 26.0 software for analysis. Group comparisons were made by analysis of variance (ANOVA) when the variables fitted the normal distribution, and Post Hoc test were conducted by Least Significant Difference (LSD) t-test for those meet homogeneity of variance and by analysis of Games-Howell for those of heterogeneity of variance. For some variables that were not normally distributed, Kruskal–Wallis test was used. A probability level of 0.05 was used to determine statistical significance.

## Results

In comparison at T1, NA-PA (*P* = 0.004), Go-Pog (*P* = 0.000), Co-Pog (*P* = 0.003), ANB (*P* = 0.006), Vertical Ratio (*P* = 0.000) and U6-PP (*P* = 0.007) showed between-group differences (Table 2).NA-PA, Go-Pog, Co-Pog and ANB were comparable for MA group and Vanbeek, Herbst and Twin-Block groups. Vertical Ratio and U6-PP of MA group differed significantly from the other groups.

**Table 2:** Descriptive statistics and group comparison at T1

Variable	Vanbeek	Herbst	Twin-Block	MA	Control	*P*
	Mean ± SD	Mean ± SD	Mean ± SD	Mean ± SD	Mean ± SD	
SNA	82.93 ± 2.00	81.02 ± 2.13	81.71 ± 2.40	81.44 ± 3.31	81.06 ± 3.07	0.346
SNB	75.94 ± 1.80	73.66 ± 3.37	74.08 ± 2.34	75.72 ± 3.24	74.83 ± 2.68	0.172
FH-NP	83.63 ± 3.30	83.63 ± 4.49	83.25 ± 2.79	84.36 ± 3.01	84.53 ± 2.62	0.849
NA-PA^†^	15.65/3.70	19.10/11.10	16.25/4.70	11.55/3.60	13.60/4.90	0.004*
MP-FH	30.31 ± 4.15	27.62 ± 7.09	29.71 ± 5.85	26.70 ± 5.16	29.67 ± 4.38	0.356
MP-SN^†^	35.65/6.40	35.50/10.0	33.80/9.60	35.05/4.60	36.40/4.60	0.847
Co-Go	52.87 ± 5.43	55.34 ± 6.10	54.18 ± 4.84	52.83 ± 3.67	53.52 ± 4.16	0.696
Go-Pog	59.59 ± 4.87	57.36 ± 4.71	57.14 ± 3.97	69.20 ± 3.33	56.50 ± 3.22	0.000*
Co-Pog	95.83 ± 6.48	95.27 ± 5.91	94.58 ± 5.27	102.14 ± 4.06	93.96 ± 4.83	0.003*
Y Axis Angle	72.54 ± 2.33	73.30 ± 3.86	72.68 ± 2.29	71.71 ± 3.06	73.57 ± 2.57	0.509
ANB^†^	7.20/1.60	7.70/3.70	7.80/1.40	5.80/1.40	6.05/1.60	0.006*
Lower Facial Height Ratio^†^	53.60/2.3	52.40/2.90	53.05/3.30	53.25/3.80	53.75/3.9	0.302
Vertical Ratio^†^	1.15/0.10	1.10/0.10	1.10/0.10	1.50/0.20	1.10/0.40	0.000*
P-A Face Height^†^	63.95/4.80	64.40/10.70	65.70/8.00	63.25/2.30	63.35/3.00	0.916
U1-SN	110.39 ± 6.37	109.81 ± 7.79	110.33 ± 7.43	107.61 ± 6.74	107.37 ± 6.53	0.671
U1-PP^†^	28.35/2.30	27.70/5.00	26.30/4.20	27.65/3.30	27.45/4.30	0.634
U6-PP	18.80 ± 3.31	20.11 ± 2.21	19.01 ± 2.16	16.58 ± 2.12	19.28 ± 1.83	0.007*
L1-MP	99.37 ± 2.98	99.34 ± 5.80	97.89 ± 8.58	97.23 ± 6.28	97.95 ± 6.66	0.874
U1-L1	113.10 ± 8.08	115.17 ± 8.22	114.84 ± 9.83	120.36 ± 7.37	116.48 ± 7.09	0.201
OP-FH	12.94 ± 3.64	11.53 ± 6.24	14.89 ± 3.58	13.99 ± 3.57	12.58 ± 4.23	0.374

^†^Non parametric test; *P < 0.05

**Fig. 2:**
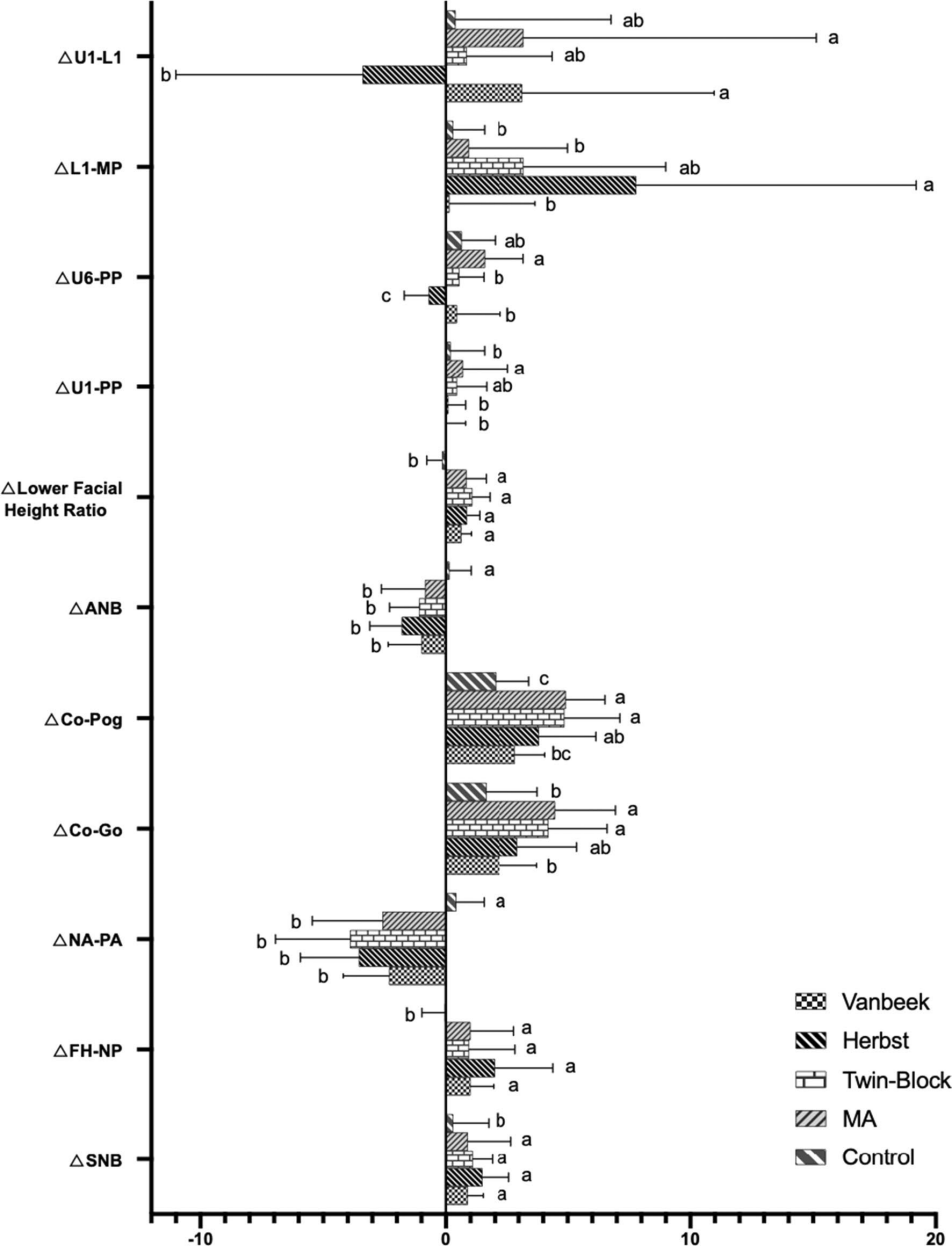
Multiple comparisons between groups from T1 to T2

The treatment changes showed significant differences in 11 variables (SNB, FH-NP, NA-PA, Co-Go, Co-Pog, ANB, lower facial height ratio, U1-PP, U6-PP, L1-MP and U1-L1) (Table 3, Fig. 2). In the four treatment groups, SNB, FH-NP and lower facial height ratio increased and NA-PA and ANB decreased, were all significantly different to the controls. In terms of the variables representing elongation of mandible, Co-Go of Twin-Block and MA groups increased significantly more than Vanbeek and control groups, meanwhile Co-Pog of Herbst, Twin-Block and MA groups increased significantly different to the controls and that of Twin-Block and MA groups was comparable for Vanbeek group. In the case of tooth movement, MA group showed significantly different changes for U1-PP than the other groups except Twin-Block; U6-PP of Herbst group decreased significantly while the others all increased and MA group was also comparable for Vanbeek and Twin-Block groups; L1-MP of Herbst group increased greatly and was significantly different to Vanbeek, MA and control groups while that of Vanbeek Activator group decreased slightly (mean = − 0.26°); and U1-L1 for Vanbeek and MA groups increased and significantly differed from Herbst group of which U1-L1 decreased about 3.4 degree.

**Table 3:** Descriptive statistics and group comparison from T1 to T2

Variable	Vanbeek	Herbst	Twin-Block	MA	Control	P
	Mean ± SD	Mean ± SD	Mean ± SD	Mean ± SD	Mean ± SD	
SNA	− 0.11 ± 0.81	− 0.01 ± 0.65	0.12 ± 1.11	0.18 ± 0.72	0.35 ± 0.84	0.692
SNB^†^	0.90/0.63^a^	1.50/1.10^a^	1.10/0.80^a^	0.90/1.78^a^	0.30/1.45^b^	0.004*
FH-NP^†^	1.00/0.95^a^	2.00/2.40 ^a^	0.95/1.90 ^a^	1.00/1.80 ^a^	− 0.05/0.93^b^	0.003*
NA-PA	− 2.31 ± 1.87^b^	− 3.55 ± 2.38^b^	− 3.91 ± 3.03^b^	− 2.59 ± 2.85^b^	0.43 ± 1.13^a^	0.000*
MP-FH	− 0.15 ± 2.37	− 0.15 ± 1.76	0.15 ± 1.38	0.41 ± 1.42	− 0.34 ± 1.25	0.814
MP-SN	0.69 ± 2.97	− 0.17 ± 1.57	0.20 ± 1.60	0.15 ± 1.58	− 0.3 ± 1.15	0.725
Co-Go	2.21 ± 1.52^b^	2.95 ± 2.42^ab^	4.22 ± 2.39^a^	4.49 ± 2.46^a^	1.66 ± 2.09^b^	0.006*
Go-Pog	1.30 ± 0.98	1.82 ± 1.68	1.81 ± 1.58	2.25 ± 1.67	1.39 ± 1.38	0.425
Co-Pog	2.84 ± 1.23^bc^	3.84 ± 2.31^ab^	4.87 ± 2.26^a^	4.93 ± 1.59^a^	2.06 ± 1.35^c^	0.015*
Y Axis Angle^†^	− 0.20/1.13	− 0.60/1.00	0.10/0.98	0.25/0.87	0.05/0.75	0.327
ANB^†^	− 1.00/1.35^b^	− 1.80/1.30^b^	− 1.10/1.20^b^	− 0.85/1.78^b^	0.15/0.88^a^	0.000*
Lower Facial Height Ratio	0.63 ± 0.41 ^a^	0.86 ± 0.52^a^	1.08 ± 0.72^a^	0.84 ± 0.81^a^	− 0.16 ± 0.62^b^	0.000*
Vertical Ratio^†^	0.00/0.05	0.00/0.10	0.00/0.07	0.00/0.03	0.00/0.10	0.303
P-A Face Height	0.50 ± 1.50	0.98 ± 1.42	0.83 ± 1.19	0.56 ± 1.54	0.85 ± 1.12	0.891
U1-SN	− 2.80 ± 4.74	− 3.17 ± 3.90	− 3.43 ± 4.35	− 4.10 ± 6.00	0.13 ± 2.86	0.191
U1-PP^†^	0.00/0.80^b^	0.10/0.70^b^	0.45/1.22^ab^	0.70/1.85^a^	0.20/1.38^b^	0.028*
U6-PP^†^	0.45/1.80^b^	− 0.70/1.00^c^	0.55/1.00^b^	1.60/1.58^a^	0.65/1.37^ab^	0.000*
L1-MP^†^	0.15/3.52^b^	7.80/11.40^a^	3.20/5.80^ab^	0.95/4.05^b^	0.30/1.28^b^	0.027*
U1-L1^†^	3.15/7.82^a^	− 3.40/7.60^b^	0.85/3.52^ab^	3.20/11.93^a^	0.40/6.37^ab^	0.034*
OP-FH	− 0.77 ± 1.61	0.74 ± 2.22	− 0.11 ± 1.86	0.12 ± 2.05	− 0.47 ± 1.43	0.328

^†^Non parametric test. Groups with the same letter indicated no statistically significant difference. *P < 0.05

### Class II correction

**Fig. 3:**
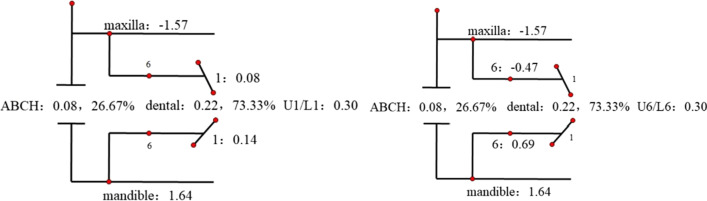
Overjet(left) and Molar Relationship (right) Analysis of C group

**Fig. 4:**
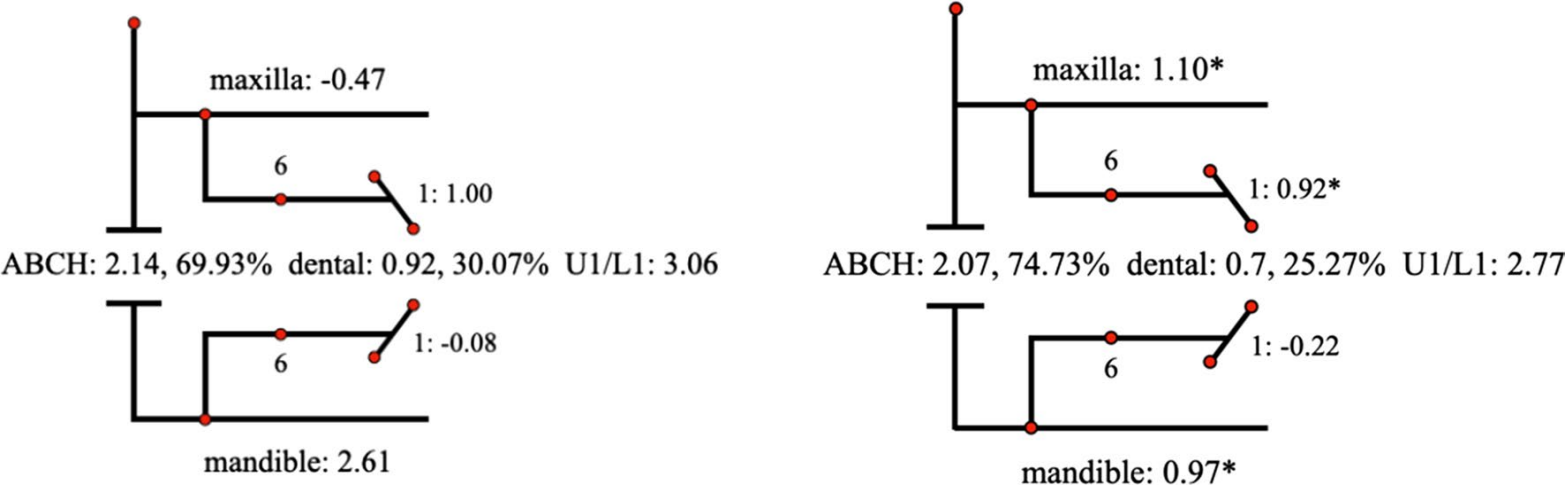
Overjet Analysis (left) and Corrected Analysis (right) of V group

**Fig. 5:**
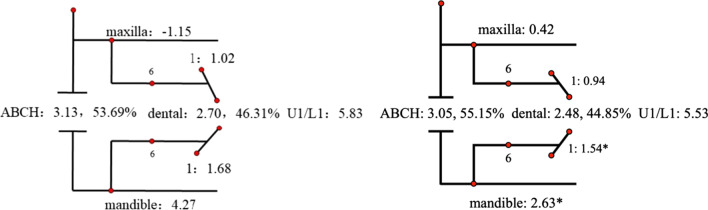
Overjet Analysis (left) and Corrected Analysis (right) of H Group

**Fig. 6:**
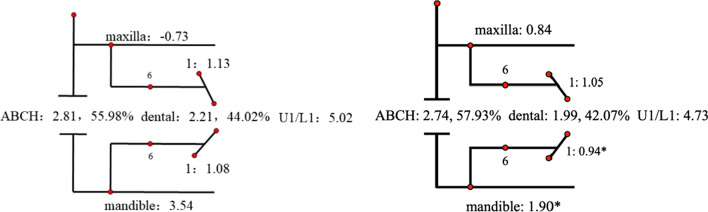
Overjet Analysis (left) and Corrected Analysis (right) of TB Group

**Fig. 7:**
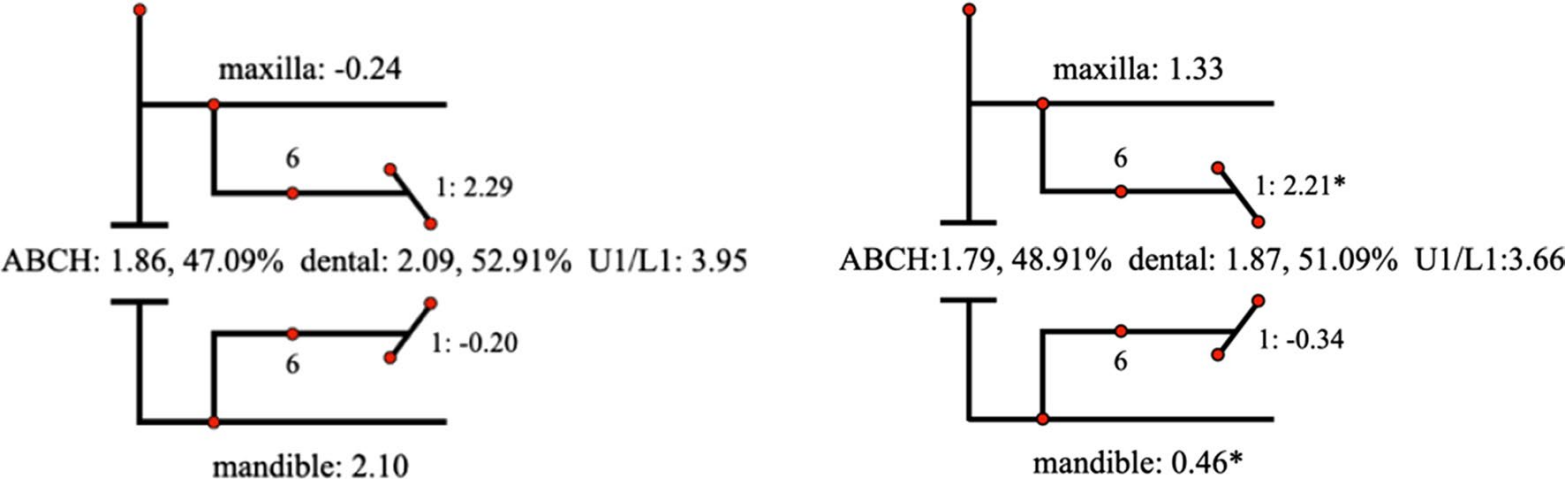
Overjet Analysis (left) and Corrected Analysis (right) of MA Group

**Fig. 8:**
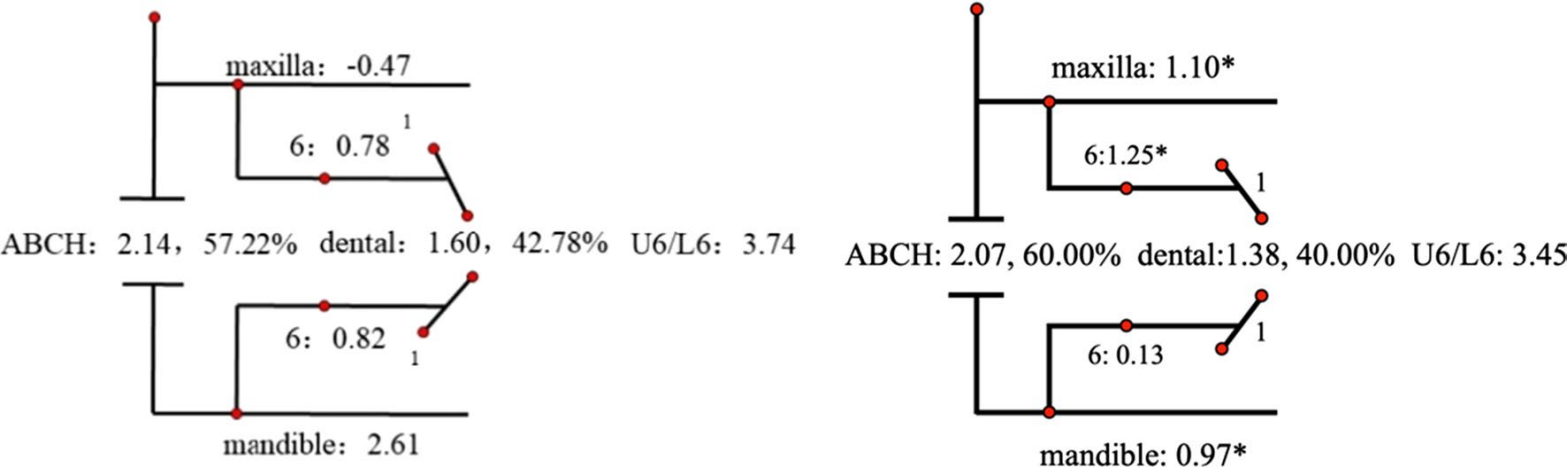
Molar Relationship Analysis (left) and Corrected Analysis (right) of V Group

**Fig. 9:**
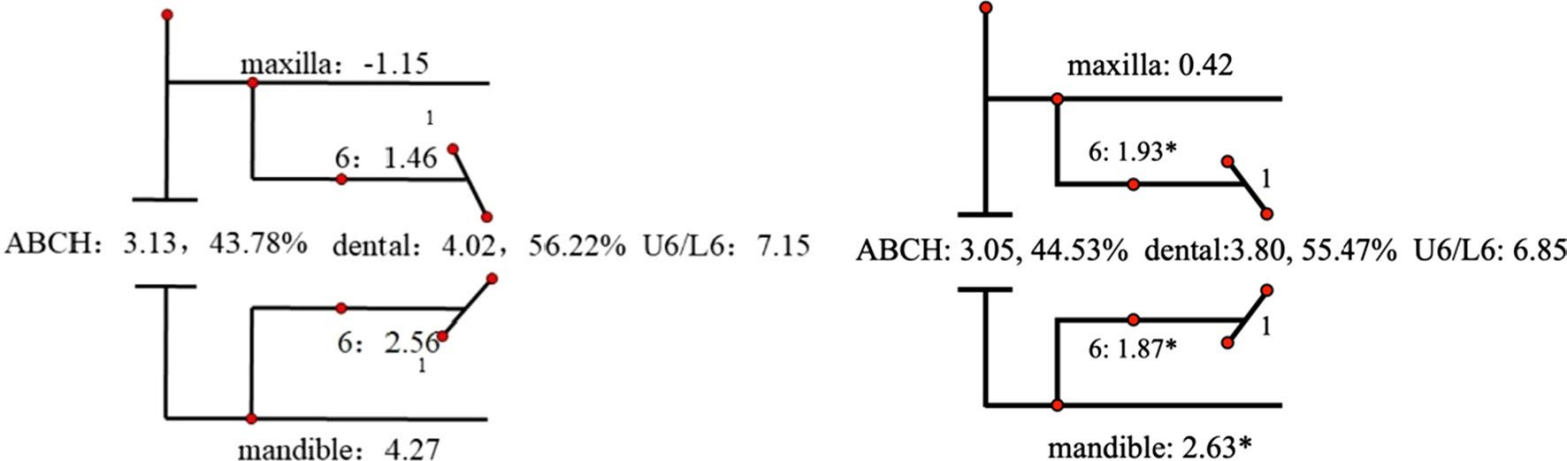
Molar Relationship Analysis (left) and Corrected Analysis (right) of H Group

**Fig. 10:**
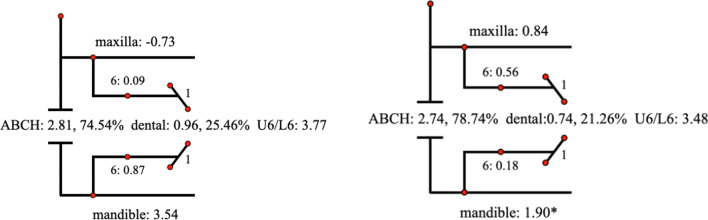
Molar Relationship Analysis (left) and Corrected Analysis (right) of TB Group

**Fig. 11:**
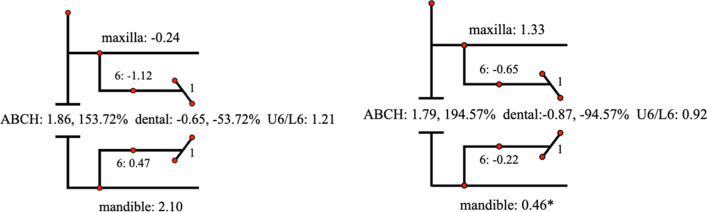
Molar Relationship Analysis (left) and Corrected Analysis (right) of MA Group

Descriptive statistics for the Johnston analysis variables are shown in Table 4, all variables except movement of maxillary osseous base were statistically different. Linear changes measured along the mean functional occlusal plane during treatment are depicted diagrammatically in the pitchforks (Fig. 3, 4, 5, 6, 7, 8, 9, 10, 11). In comparison of the four treatment groups, Herbst showed the most ABCH and Vanbeek Activator showed the least (Herbst > Twin-Block > MA > Vanbeek Activator) with no significance. Except for the MA group, the upper molars in the other groups moved positively distally and there were significant differences between MA group and Herbst & Vanbeek groups. Upper molar movement in Twin-Block group and lower one in MA and Vanbeek groups were significantly less than that in Herbst group. Lower incisor movement in MA and Vanbeek groups, which was negative, were significantly less than that in both Herbst and Twin-Block groups which were positive. Of all the groups, the MA group had the largest upper incisor movement, but it was not statistically significant.

**Table 4:** Skeletal and dental changes in molar relationship and overjet

Variable(mm)	Vanbeek	Herbst	Twin-Block	Ma	Control	P
	Median/IQR	Median/IQR	Median/IQR	Median/IQR	Median/IQR	
ABCH^†^	2.05/0.90^a^	3.60/1.40^a^	2.65/2.20^a^	2.59/3.20^a^	0.20/1.40^b^	0.000*
Maxillary Osseous base^†^	− 0.50/1.30	− 0.80/2.00	− 0.40/1.00	− 0.98/3.00	− 1.75/1.30	0.059
Upper Molar^†^	0.75/1.20^ab^	1.30/1.00^a^	0.00/1.60^bc^	− 1.58/2.80^c^	− 0.50/1.00^c^	0.000*
Lower Molar^†^	0.60/0.90^b^	2.80/2.40^a^	1.30/1.20^ab^	0.37/2.90^b^	0.40/1.50^b^	0.008*
Upper Incisor^†^	1.25/2.20^a^	0.90/0.80^ab^	0.90/1.70^ab^	2.35/2.50^a^	0.00/1.30^b^	0.011*
Lower Incisor^†^	0.00/0.90^b^	1.20/1.60^a^	1.25/1.20^a^	0.10/3.50^b^	− 0.20/1.80^b^	0.002*

^†^Non parametric test. Groups with the same letter indicated no statistically significant difference. *P < 0.05

### Skeletal and dentoalveolar effect in four appliances

The skeletal effect of Vanbeek was more important in improving the deep overjet of anterior teeth, while in MA group dental changes accounted more (Table 5). In terms of correction of class II molar occlusion, Twin-Block and MA groups accounted for more bone effects among the four appliances.

**Table 5:** Skeletal and Dentoalveolar Effect in V, H, TB, MA

Groups	Overjet correction(%)				Molar relationship correction(%)			
	Skeletal effects (%)	Skeletal effects (corrected)	Dentoalveolar effects (%)	Dentoalveolar effects (corrected)	Skeletal effects (%)	Skeletal effects (corrected)	Dentoalveolar effects (%)	Dentoalveolar effects (corrected)
Vanbeek	69.93	74.73	30.07	25.27	57.22	60.00	42.78	40.00
Herbst	53.69	55.15	46.31	44.85	43.78	44.53	56.22	55.47
Twin-Block	55.98	57.93	44.02	42.07	74.54	78.74	25.46	21.26
MA	47.09	48.91	52.91	51.09	153.72	194.57	− 53.72	− 94.57

## Discussion

As shown in the result, all the four appliances are effective in correcting class II malocclusion. Compared with the untreated group, Vanbeek Activator, Herbst, Twin-Block and MA created a significant orthopedic effect that mandible forward repositioning and facial profile improvement, which is consistent with previous studies [4, 12–14]. However, untreated children have no potential to correct class II malocclusion by themselves, so orthopedic treatment quietly benefits a lot[3, 4]. And there actually were differences in basal bone movements based on the Johnston’s Pitchfork Analysis [3, 15].

As for mandibular growth, supplementary mandibular elongation was found in Twin-Block and MA (Co-Go and Co-Pog) and Herbst (Co-Pog). This was in agreement with previous animal researches of animal models, that mandible growth was enhanced by functional appliances, especially in vertical direction [16, 17]. DAnto’s systematical review found that most studies reported significant mandibular length increasing when treated at the adolescent growth spurt [12], and Sabouni et.al also confirmed that the same result could be obtained after MA treatment [10]. Phan believed that fixed nature of Herbst made its action time longer than others, which brought about more mandibular length growth [18]. However, Johnston’s research didn’t support the point mentioned above [19].

Although with a lack of significant reduction of SNA in the four treatment groups, maxilla growth inhibition was observed in Vanbeek Activator via maxilla base bone movement in Pitchfork Analysis, which also suggested that the torque of upper incisor might interfere the position of A point and our distinguishment between dental and skeletal effects [3, 20]. This was consisted with previous studies that headgear had some effects in maxillary restrain [4, 21, 22]. However clinically significant restraint of maxillary growth was not clear in other functional appliances [23, 24]. Some studies have reported maxillary inhibition with Twin-Block and Herbst, but others supposed that was negligible, which might come from changes in A point.

Compared with controls, the four treatment groups all significantly increased the lower facial height and worsen the long face profile. That might come from the mandibular clockwise rotation during occlusion reconstruction [25]. Activator was reported a significant increase in the lower facial height, which was primarily because of the bite opening effect and guided extrusion of upper molars [26]. Vanbeek Activator presented the least increase in lower facial height among the four appliances since the high-pull headgear inhibited the downward growth of maxilla and resulted in the slightest mandible clockwise rotation, which was consistent with Spalj’s observation[4]. Brien’s research found that the high-pull headgear could make the mandible rotate forward [15], and the finite element analysis results of Gautam et al. also suggested that it could effectively carry out vertical control[27]. In addition, changes in vertical dimension were also related to molars’ vertical movements. Herbst and clear aligners were reported to give rise to molar intrusion [28, 29], which offset some mandibular clockwise rotation caused by bite opening effect. Even some studies observed a mandibular counterclockwise rotation in Herbst [30]. However, in current research, Herbst and MA didn’t induce any significant change in the inclination of the mandibular plane to the Frankfort horizontal plane or to SN plane, as also confirmed in Caruso’s research [7].

From our results, Activator, Herbst and Twin-Block had different levels of upper incisor retroclination and lower incisor proclination [4, 31–33], but decrease in upper incisor/Sella-Nasion plane angle (U1-SN) didn’t present significant difference. L1-MP in Herbst increased significantly more than that in Vanbeek Activator and MA. Herbst exerts the force directly on teeth without any effective control for lower incisors, except a lingual bar, thus produces the most evident labial movement as a result[31]. The headgear activator could effectively control the inclination of lower anterior teeth[25, 34]. In this study, Vanbeek appliance showed a slight proclination which was attributed to the counterclockwise rotation of lower incisors under the headgear’s extraoral forces in backward direction [34, 35]. That is consistent with some previous studies that inclination of lower incisors in Activator patients almost remain the same or changes a little [36]. Different from other three appliances, MA provided us with digital means to design the movement of teeth and the then produced corresponding aligners via 3D printing technology. Attributed to its surrounding of each teeth crown surface we could control orthodontic tooth movement while moving the mandible forward [7, 8]. Sabouni et.al reported the lower incisor angulation was maintained during class II correction [10]. In this sample, MA presents little compensatory proclination of lower incisors, which could provide more space for appliances to play the skeletal role of guiding the mandible forward.

As mentioned before, point A and B, which are broadly used as skeletal marks of maxilla and mandible, are likely affected by torque of upper and lower incisors [3]. In this way the real skeletal effects of functional appliances are easily biased by dental movements. Compared with conventional cephalometrics, Johnston’s Pitchfork Analysis is a better method to analyze skeletal and dentoalveolar changes separately and calculate their proportions [37].

Although all the four appliances greatly corrected molar relationship and decreased overjet, their effects were distinctly different. At the level of overjet reduction, Vanbeek activator accounted the highest proportion of skeletal effect (74.73%), which was consistent with previous researches [25, 38]. This could be owing to its structure characteristics that it entirely wrapped the whole upper and lower arch and high-pull headgear inhibited maxillary forward and vertical growth. For the MA group, Ravera et al. reported that if the patients were treated at CVM2 growth phase, the aligners showed mainly dentoalveolar effects while at CVM3 the skeletal components accounted for more [8], which was consistent with our results. Although dental effect shared higher proportion (51.09%), bone effect was still close to a half (48.91%). Blackham also supported that skeletally MA didn’t present outcomes significantly different from the Twin-Block [14]. Conversely, MA was more comfortable, less noticeable and easier to insert than TB, which could improve the degree of compliance [39]. Additionally clear aligners could realize the design of terminal occlusion in advance and have good control of teeth movement, which overcomes the common recognized limit of Class II conventional functional appliances and allow an optimization of clinical efficacy [7, 40]. Dental changes accounted for 44.85% and 42.07% in Herbst and Twin-Block in this sample. While the existing literature data, ranging from 40 to 65% [41–44], also have some differences on the weight of dentoalveolar effects caused by Herbst or Twin-Block, which may be due to the difference in the age of their cases.

At the end of treatment, the molar relationship can be improved from the distal occlusal relationship to the neutral or even slightly mesial relationship [41, 45], and some of these improvements are contributed to the dental movement of the molars. In terms of molar relationship correction of this sample, the proportions of dentoalveolar effects are 40.00%, 55.47%, 21.26% and − 94.57% in Vanbeek Activator, Herbst, Twin-Block and MA. Lagerstrom et al. found that distal movement of the maxillary molars was almost entirely dental and mandibular advancement devoted most of lower molar mesial movement in Headgear-Activator treatment [46]. Jena et al. found that after Twin-Block treatment, the relationship of the molars that were originally distal was significantly corrected and had significant distal movement of the maxillary molars and proximal movement of the mandibular molars [47]. Several studies found that Herbst has a significant dental effect of pushing the maxillary molars backward [41, 48], and Tomblyn’s research on fixed Herbst showed that 60% of the molar relationship change was the molar movement factor [41, 44]. The above conclusions were basically consistent with the results of this study. MA adjusted the movement of the molars to establish a neutral occlusal relationship after several “bite jump” mandibular advancements. The maxillary molars moved mesially to adapt to the position of the mandibular molars after the mandibular lead, and MA enabled to distalize the lower molars, to establish a neutral molar relationship. Any distalization would result in a net negative movement with respect to initial maxillary molar position relative to the occlusal plane [14], as is shown in our sample (− 94.57% for dentoalveolar effect). Typically, children with class II malocclusion are associated with narrow arches, thus sometimes the upper arch expansion guided by the aligners, in order to create more room for alignment, leveling and the forwarded position of the mandible, can meanwhile promote posterior teeth buccal tipping as resulted[49].

The present results are limited by the small number of patients, the retrospective design and lack of MRI or CBCT to assess TMJ conditions. For this reason, further studies with a longitudinal randomized design on a larger sample and the changes of temporomandibular joint region are encouraged.

### Strengths and limitations

The strength of this study is to compare the therapeutic effect of the clear aligners with the traditional one via both cephalometric analysis and Johnston Pitchfork analysis. On the other hand, due to the retrospective nature of this study, only limited data analysis could be performed with existing patient recordings. In addition, the number of patients enrolled in this study was small due to the single-center trial, with only more than 10 cases in each group, and no comparative analysis was conducted with other orthotics.

## Conclusions

1. Four appliances are all effective in mandibular advancement, facial profile improvement, skeletal class II correction and modification of class II molar relationship and deep overjet, with unavoidable increase in lower facial ratio, however.
2. Vanbeek shows the highest proportion of skeletal changes, more significant maxillary growth inhibition and better control of lower incisor.
3. Herbst corrects the overjet of Class II patients more by skeletal changes accompanied with lower incisors proclination and has greater maxillary molar distalization.
4. Twin-Block shows lower incisors proclination and presents both skeletal and dentoalveolar effects.
5. MA corrects the overjet of Class II patients more by dentoalveolar effect. MA allows aligning and leveling to establish a good occlusal relationship while leading the mandible forward, which may save time for phase II orthodontics, and has good control of incisor inclination and enables to distalize molars.

## Data Availability

The data underlying this article will be shared on reasonable request to the corresponding author.
